# 2-(4-Hydroxy­biphenyl-3-yl)isoindolin-1-one

**DOI:** 10.1107/S1600536810002370

**Published:** 2010-01-27

**Authors:** Yu Zheng, Jin-Long Wu

**Affiliations:** a Laboratory of Asymmetric Catalysis and Synthesis, Department of Chemistry, Zhejiang University, Hangzhou 310027, People’s Republic of China

## Abstract

In the mol­ecular structure of the title compound, C_20_H_15_NO_2_, the isoindolin-1-one unit is planar, the maximum atomic deviation being 0.048 (2) Å. The two biphenyl rings are twisted with respect to the isoindolin-1-one plane, making dihedral angles of 33.21 (9) and 33.34 (9)°. The two benzene rings of the biphenyl substituent are oriented at a dihedral angle of 35.43 (11)° to each other. An intra­molecular O—H⋯O inter­action occurs and inter­molecular C—H⋯O hydrogen bonding is present in the crystal structure.

## Related literature

For the biological activity of isoindolin-1-ones, see: Nozawa *et al.* (1997 ▶); Atack *et al.* (2006 ▶); Lunn *et al.* (2004 ▶). For the reaction conditions for the synthesis of the title compound, see: Wu *et al.* (2007 ▶). For the palladium-catalysed intra­molecular deca­rbonylative coupling mechanism, see: Baudoin (2007 ▶).
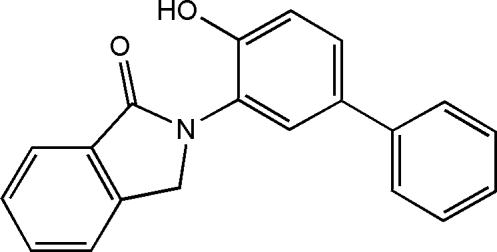


## Experimental

### 

#### Crystal data

C_20_H_15_NO_2_
*M* *_r_* = 301.33
Tetragonal, 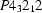

*a* = 7.5123 (2) Å
*c* = 52.3543 (17) Å
*V* = 2954.60 (15) Å^3^
*Z* = 8
Cu *K*α radiation
μ = 0.70 mm^−1^
*T* = 294 K
0.32 × 0.22 × 0.20 mm

#### Data collection

Rigaku R-AXIS RAPID IP diffractometer
Absorption correction: multi-scan (*ABSCOR*; Higashi, 1995 ▶) *T*_min_ = 0.822, *T*_max_ = 0.900
10482 measured reflections
1676 independent reflections
1556 reflections with *I* > 2σ(*I*)
*R*_int_ = 0.041

#### Refinement

*R*[*F* ^2^ > 2σ(*F* ^2^)] = 0.031
*wR*(*F* ^2^) = 0.077
*S* = 1.08
1676 reflections
210 parameters
H-atom parameters constrained
Δρ_max_ = 0.16 e Å^−3^
Δρ_min_ = −0.15 e Å^−3^

### 

Data collection: *PROCESS-AUTO* (Rigaku, 1998 ▶); cell refinement: *PROCESS-AUTO*; data reduction: *CrystalStructure* (Rigaku/MSC, 2002 ▶); program(s) used to solve structure: *SIR92* (Altomare *et al.*, 1993 ▶); program(s) used to refine structure: *SHELXL97* (Sheldrick, 2008 ▶); molecular graphics: *ORTEP-3 for Windows* (Farrugia, 1997 ▶); software used to prepare material for publication: *WinGX* (Farrugia, 1999 ▶).

## Supplementary Material

Crystal structure: contains datablocks global, I. DOI: 10.1107/S1600536810002370/xu2719sup1.cif

Structure factors: contains datablocks I. DOI: 10.1107/S1600536810002370/xu2719Isup2.hkl

Additional supplementary materials:  crystallographic information; 3D view; checkCIF report

## Figures and Tables

**Table 1:** Hydrogen-bond geometry (Å, °)

*D*—H⋯*A*	*D*—H	H⋯*A*	*D*⋯*A*	*D*—H⋯*A*
O2—H2⋯O1	0.82	1.79	2.575 (2)	162
C20—H20⋯O1^i^	0.93	2.37	3.283 (3)	168

Symmetry code: (i) 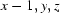.

**Fig. 1.**
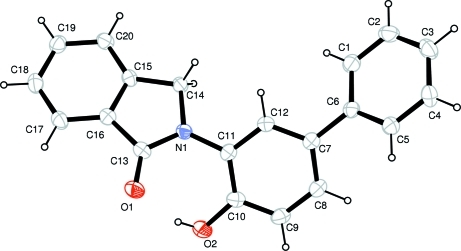
The molecular structure of (I). Displacement ellipsoids are drawn at 40% probability level and H atoms are shown as small circles of arbitary radii.

## supplementary crystallographic information

### Comment

The title compound is a derivative of isoindolin-1-ones, which has been reported to deliver various biological activities such as neuritogenic activity (Nozawa *et al.*,  1997); anxiolytic activity (Atack *et al.*,  2006), survival motor neuron (SMN)-reporter upregulation activity (Lunn *et al.*, 2004). In our laboratory the crystals of title compound was obtained unexpectedly when 4-(2'-bromobenzyl)-6-phenylbenz[1,4]oxazine-2,3-dione was subjected to the palladium-catalyzed intramolecular direct arylation (Wu *et al.*,  2007). It seems that the substrate underwent an intramolecular decarbonylative coupling under the palladium-catalyzed conditions (Baudoin, 2007). The structure of the title compound has been characterized by spectroscopic methods with further confirmation by X-ray analysis. We report here its crystal strutture.

In the molecular structure of the title compound, there are one biphenyl moiety and one isoindolin-1-one linked through C11—N1 single bond (Fig. 1). The isoindolin-1-one moiety has a coplanar structure, the maximum atomic deviation being 0.048 (2) Å. Two phenyl rings are twisted with respect to the isoindolin-1-one plane with dihedral angles of 33.21 (9) and 33.34 (9)°, respectively. The two phenyl rings are oriented at 35.43 (11)°. The O—H···O and C—H···O hydrogen bonding is present in the crystal structure (Table 1).

### Experimental

A 10 mL flask was charged with Pd(OAc)_2_ (6.7 mg, 0.03 mmol), 1,1'-bis(diphenylphosphino)ferrocene (dppf) (16.6 mg, 0.03 mmol), and K_2_CO_3_ (83.0 mg, 0.6 mmol). The loaded flask was evacuated and backfilled with N_2_ (repeated for three times). To the degassed flask was added a solution of 4-(2'-bromobenzyl)-6-phenylbenz[1,4]oxazine-2,3-dione (122.4 mg, 0.3 mmol) in degassed DMA (3 mL). The resultant mixture was heated at 393 K for 2 h under a nitrogen atmosphere. After cooling to room temperature, the reaction was quenched by adding CH_2_Cl_2_ (20 mL), and the resultant mixture was washing with H_2_O (3 × 10 mL) to remove DMA. The organic layer was washed with brine, dried over anhydrous Na_2_SO_4_, filtrated, and concentrated under reduced pressure. The residue was purified by column chromatography over silica gel with elution by 20% EtOAc in petroleum ether (333–363 K) to give 2-(4'-hydroxybiphenyl-3'-yl)isoindolin- 1-one (70.0 mg, 78%), m.p. 453–454 K (CH_2_Cl_2_-hexane). Single crystals suitable for X-ray diffraction of the title compound were grown in the mixed solvent of CH_2_Cl_2_ and hexane.

### Refinement

H atoms were placed in calculated positions with C—H = 0.93–0.97 and O—H = 0.82 Å, and included in the refinement in riding model with *U*_iso_(H) = 1.2*U*_eq_(C) and 1.5U_eq_(O). As no significant anomalous scattering effects were observed, Friedel pairs were merged.

### Crystal data

**Table:**

C_20_H_15_NO_2_	*D*_x_ = 1.355 Mg m^−^^3^
*M**_r_* = 301.33	Cu *K*α radiation, λ = 1.54184 Å
Tetragonal, *P*4_3_2_1_2	Cell parameters from 3246 reflections
Hall symbol: P 4nw 2abw	θ = 3.5–65.0°
*a* = 7.5123 (2) Å	µ = 0.70 mm^−^^1^
*c* = 52.3543 (17) Å	*T* = 294 K
*V* = 2954.60 (15) Å^3^	Prism, colorless
*Z* = 8	0.32 × 0.22 × 0.20 mm
*F*(000) = 1264	

### Data collection

**Table:**

Rigaku R-AXIS RAPID IP diffractometer	1676 independent reflections
Radiation source: fine-focus sealed tube	1556 reflections with *I* > 2σ(*I*)
graphite	*R*_int_ = 0.041
Detector resolution: 10.0 pixels mm^-1^	θ_max_ = 67.1°, θ_min_ = 3.4°
ω scans	*h* = −8→8
Absorption correction: multi-scan (*ABSCOR*; Higashi, 1995)	*k* = −8→8
*T*_min_ = 0.822, *T*_max_ = 0.900	*l* = −56→61
10482 measured reflections	

### Refinement

**Table:**

Refinement on *F*^2^	Secondary atom site location: difference Fourier map
Least-squares matrix: full	Hydrogen site location: inferred from neighbouring sites
*R*[*F*^2^ > 2σ(*F*^2^)] = 0.031	H-atom parameters constrained
*wR*(*F*^2^) = 0.077	*w* = 1/[σ^2^(*F*_o_^2^) + (0.0383*P*)^2^ + 0.7354*P*] where *P* = (*F*_o_^2^ + 2*F*_c_^2^)/3
*S* = 1.08	(Δ/σ)_max_ = 0.001
1676 reflections	Δρ_max_ = 0.16 e Å^−^^3^
210 parameters	Δρ_min_ = −0.15 e Å^−^^3^
0 restraints	Extinction correction: *SHELXL97* (Sheldrick, 2008), Fc^*^=kFc[1+0.001xFc^2^λ^3^/sin(2θ)]^-1/4^
Primary atom site location: structure-invariant direct methods	Extinction coefficient: 0.00108 (16)

### Special details

**Table:**

Geometry. All e.s.d.'s (except the e.s.d. in the dihedral angle between two l.s. planes) are estimated using the full covariance matrix. The cell e.s.d.'s are taken into account individually in the estimation of e.s.d.'s in distances, angles and torsion angles; correlations between e.s.d.'s in cell parameters are only used when they are defined by crystal symmetry. An approximate (isotropic) treatment of cell e.s.d.'s is used for estimating e.s.d.'s involving l.s. planes.
Refinement. Refinement of *F*^2^ against ALL reflections. The weighted *R*-factor *wR* and goodness of fit *S* are based on *F*^2^, conventional *R*-factors *R* are based on *F*, with *F* set to zero for negative *F*^2^. The threshold expression of *F*^2^ > σ(*F*^2^) is used only for calculating *R*-factors(gt) *etc*. and is not relevant to the choice of reflections for refinement. *R*-factors based on *F*^2^ are statistically about twice as large as those based on *F*, and *R*- factors based on ALL data will be even larger.

### Fractional atomic coordinates and isotropic or equivalent isotropic displacement parameters (Å^2^)

**Table:**

	*x*	*y*	*z*	*U*_iso_*/*U*_eq_	
O1	1.19213 (19)	0.1923 (2)	0.94864 (3)	0.0332 (4)	
O2	1.2708 (2)	−0.0751 (2)	0.92001 (2)	0.0343 (4)	
H2	1.2642	0.0021	0.9310	0.052*	
N1	0.9322 (2)	0.1133 (2)	0.92751 (3)	0.0227 (4)	
C1	0.6483 (3)	0.0856 (3)	0.83609 (4)	0.0298 (5)	
H1	0.5924	0.0596	0.8515	0.036*	
C2	0.5463 (3)	0.1296 (3)	0.81496 (4)	0.0349 (5)	
H2A	0.4229	0.1325	0.8163	0.042*	
C3	0.6272 (3)	0.1694 (3)	0.79190 (4)	0.0348 (5)	
H3	0.5589	0.2004	0.7778	0.042*	
C4	0.8105 (3)	0.1625 (3)	0.79011 (3)	0.0326 (5)	
H4	0.8655	0.1875	0.7746	0.039*	
C5	0.9131 (3)	0.1186 (3)	0.81118 (3)	0.0282 (5)	
H5	1.0364	0.1150	0.8097	0.034*	
C6	0.8337 (3)	0.0797 (3)	0.83461 (3)	0.0239 (4)	
C7	0.9453 (3)	0.0361 (3)	0.85723 (3)	0.0242 (4)	
C8	1.1044 (3)	−0.0590 (3)	0.85490 (3)	0.0282 (5)	
H8	1.1399	−0.1008	0.8390	0.034*	
C9	1.2093 (3)	−0.0914 (3)	0.87605 (4)	0.0297 (5)	
H9	1.3153	−0.1539	0.8741	0.036*	
C10	1.1605 (3)	−0.0328 (3)	0.90024 (3)	0.0266 (5)	
C11	0.9992 (3)	0.0590 (3)	0.90309 (3)	0.0232 (4)	
C12	0.8957 (3)	0.0937 (3)	0.88161 (3)	0.0231 (4)	
H12	0.7902	0.1572	0.8835	0.028*	
C13	1.0282 (3)	0.1716 (3)	0.94795 (3)	0.0252 (4)	
C14	0.7394 (3)	0.1129 (3)	0.93267 (3)	0.0232 (4)	
H14A	0.6893	−0.0053	0.9306	0.028*	
H14B	0.6770	0.1950	0.9215	0.028*	
C15	0.7311 (3)	0.1730 (3)	0.96011 (3)	0.0224 (4)	
C16	0.9020 (3)	0.2040 (3)	0.96878 (3)	0.0236 (4)	
C17	0.9354 (3)	0.2596 (3)	0.99368 (3)	0.0282 (5)	
H17	1.0510	0.2791	0.9994	0.034*	
C18	0.7913 (3)	0.2848 (3)	1.00959 (3)	0.0295 (5)	
H18	0.8097	0.3196	1.0264	0.035*	
C19	0.6183 (3)	0.2586 (3)	1.00072 (4)	0.0289 (5)	
H19	0.5228	0.2788	1.0116	0.035*	
C20	0.5861 (3)	0.2026 (3)	0.97573 (3)	0.0272 (4)	
H20	0.4706	0.1857	0.9698	0.033*	

### Atomic displacement parameters (Å^2^)

**Table:**

	*U*^11^	*U*^22^	*U*^33^	*U*^12^	*U*^13^	*U*^23^
O1	0.0203 (8)	0.0453 (10)	0.0340 (7)	−0.0038 (7)	−0.0027 (6)	−0.0023 (7)
O2	0.0300 (8)	0.0425 (10)	0.0305 (7)	0.0109 (7)	−0.0058 (6)	0.0042 (6)
N1	0.0195 (8)	0.0270 (9)	0.0216 (7)	−0.0004 (7)	−0.0002 (6)	0.0013 (6)
C1	0.0282 (11)	0.0328 (12)	0.0283 (9)	−0.0004 (9)	0.0017 (8)	−0.0037 (9)
C2	0.0233 (11)	0.0391 (13)	0.0423 (11)	0.0032 (9)	−0.0050 (9)	−0.0101 (10)
C3	0.0365 (13)	0.0347 (13)	0.0332 (10)	0.0021 (10)	−0.0129 (9)	−0.0059 (9)
C4	0.0382 (13)	0.0369 (13)	0.0226 (9)	−0.0042 (10)	−0.0041 (9)	−0.0032 (9)
C5	0.0257 (11)	0.0323 (12)	0.0265 (9)	−0.0009 (9)	−0.0011 (8)	−0.0033 (8)
C6	0.0245 (10)	0.0211 (10)	0.0260 (9)	0.0004 (8)	−0.0004 (8)	−0.0057 (8)
C7	0.0247 (11)	0.0224 (10)	0.0255 (9)	−0.0019 (8)	0.0021 (8)	−0.0001 (8)
C8	0.0296 (11)	0.0277 (11)	0.0274 (9)	0.0031 (10)	0.0052 (8)	−0.0017 (8)
C9	0.0265 (12)	0.0291 (11)	0.0336 (9)	0.0065 (9)	0.0043 (9)	0.0014 (9)
C10	0.0226 (11)	0.0268 (11)	0.0302 (9)	0.0014 (8)	0.0004 (8)	0.0042 (8)
C11	0.0246 (10)	0.0218 (10)	0.0232 (9)	−0.0019 (8)	0.0031 (7)	0.0013 (8)
C12	0.0204 (10)	0.0231 (10)	0.0259 (9)	0.0004 (8)	0.0005 (7)	0.0000 (8)
C13	0.0233 (11)	0.0255 (11)	0.0268 (9)	−0.0021 (8)	−0.0037 (8)	0.0032 (8)
C14	0.0208 (10)	0.0281 (10)	0.0206 (8)	−0.0015 (8)	−0.0006 (7)	0.0010 (7)
C15	0.0265 (11)	0.0205 (10)	0.0202 (8)	−0.0014 (8)	−0.0026 (7)	0.0035 (7)
C16	0.0244 (10)	0.0220 (10)	0.0245 (8)	−0.0004 (8)	−0.0009 (8)	0.0014 (8)
C17	0.0292 (11)	0.0268 (11)	0.0286 (9)	−0.0015 (9)	−0.0072 (8)	−0.0027 (8)
C18	0.0388 (12)	0.0278 (11)	0.0218 (8)	0.0020 (9)	−0.0041 (8)	−0.0015 (8)
C19	0.0303 (11)	0.0313 (12)	0.0253 (8)	0.0021 (9)	0.0029 (8)	0.0000 (9)
C20	0.0234 (11)	0.0319 (12)	0.0263 (8)	−0.0008 (9)	−0.0012 (8)	0.0032 (8)

### Geometric parameters (Å, °)

**Table:**

O1—C13	1.242 (3)	C8—H8	0.9300
O2—C10	1.363 (2)	C9—C10	1.390 (3)
O2—H2	0.8200	C9—H9	0.9300
N1—C13	1.363 (2)	C10—C11	1.402 (3)
N1—C11	1.433 (2)	C11—C12	1.393 (3)
N1—C14	1.474 (3)	C12—H12	0.9300
C1—C2	1.386 (3)	C13—C16	1.466 (3)
C1—C6	1.396 (3)	C14—C15	1.507 (2)
C1—H1	0.9300	C14—H14A	0.9700
C2—C3	1.384 (3)	C14—H14B	0.9700
C2—H2A	0.9300	C15—C20	1.380 (3)
C3—C4	1.381 (3)	C15—C16	1.382 (3)
C3—H3	0.9300	C16—C17	1.392 (3)
C4—C5	1.386 (3)	C17—C18	1.379 (3)
C4—H4	0.9300	C17—H17	0.9300
C5—C6	1.395 (3)	C18—C19	1.394 (3)
C5—H5	0.9300	C18—H18	0.9300
C6—C7	1.487 (3)	C19—C20	1.396 (3)
C7—C8	1.398 (3)	C19—H19	0.9300
C7—C12	1.398 (3)	C20—H20	0.9300
C8—C9	1.381 (3)		
			
C10—O2—H2	109.5	C12—C11—C10	119.27 (17)
C13—N1—C11	127.30 (17)	C12—C11—N1	118.10 (17)
C13—N1—C14	112.13 (15)	C10—C11—N1	122.59 (16)
C11—N1—C14	120.56 (15)	C11—C12—C7	122.03 (18)
C2—C1—C6	121.00 (19)	C11—C12—H12	119.0
C2—C1—H1	119.5	C7—C12—H12	119.0
C6—C1—H1	119.5	O1—C13—N1	126.02 (18)
C3—C2—C1	120.3 (2)	O1—C13—C16	126.79 (18)
C3—C2—H2A	119.8	N1—C13—C16	107.19 (17)
C1—C2—H2A	119.8	N1—C14—C15	102.41 (15)
C4—C3—C2	119.3 (2)	N1—C14—H14A	111.3
C4—C3—H3	120.4	C15—C14—H14A	111.3
C2—C3—H3	120.4	N1—C14—H14B	111.3
C3—C4—C5	120.6 (2)	C15—C14—H14B	111.3
C3—C4—H4	119.7	H14A—C14—H14B	109.2
C5—C4—H4	119.7	C20—C15—C16	120.77 (16)
C4—C5—C6	120.8 (2)	C20—C15—C14	130.21 (18)
C4—C5—H5	119.6	C16—C15—C14	108.99 (16)
C6—C5—H5	119.6	C15—C16—C17	121.75 (18)
C5—C6—C1	117.99 (18)	C15—C16—C13	109.17 (15)
C5—C6—C7	120.34 (18)	C17—C16—C13	129.07 (19)
C1—C6—C7	121.66 (18)	C18—C17—C16	117.72 (19)
C8—C7—C12	117.76 (17)	C18—C17—H17	121.1
C8—C7—C6	121.68 (16)	C16—C17—H17	121.1
C12—C7—C6	120.54 (18)	C17—C18—C19	120.76 (17)
C9—C8—C7	120.55 (17)	C17—C18—H18	119.6
C9—C8—H8	119.7	C19—C18—H18	119.6
C7—C8—H8	119.7	C18—C19—C20	121.11 (19)
C8—C9—C10	121.62 (19)	C18—C19—H19	119.4
C8—C9—H9	119.2	C20—C19—H19	119.4
C10—C9—H9	119.2	C15—C20—C19	117.84 (19)
O2—C10—C9	117.24 (18)	C15—C20—H20	121.1
O2—C10—C11	123.97 (17)	C19—C20—H20	121.1
C9—C10—C11	118.74 (18)		

### Hydrogen-bond geometry (Å, °)

**Table:**

*D*—H···*A*	*D*—H	H···*A*	*D*···*A*	*D*—H···*A*
O2—H2···O1	0.82	1.79	2.575 (2)	162
C20—H20···O1^i^	0.93	2.37	3.283 (3)	168

Symmetry codes: (i) *x*−1, *y*, *z*.
